# Desalination Technology in South Korea: A Comprehensive Review of Technology Trends and Future Outlook

**DOI:** 10.3390/membranes12020204

**Published:** 2022-02-09

**Authors:** Jongkwan Park, Sungyun Lee

**Affiliations:** 1 School of Civil, Environmental and Chemical Engineering, Changwon National University, 20 Changwondaehak-ro, Changwon-si 51140, Korea; jkpark2019@changwon.ac.kr; 2 Department of Civil Environmental Engineering, School of Disaster Prevention and Environmental Engineering, Kyungpook National University, 2559 Gyeongsang-daero, Sangju-si 37224, Korea; 3 Department of Advanced Science and Technology Convergence, Kyungpook National University, 2559 Gyeongsang-daero, Sangju-si 37224, Korea

**Keywords:** seawater desalination, reverse osmosis, industry, research, alternative technology, energy, South Korea

## Abstract

Due to advances in desalination technology, desalination has been considered as a practical method to meet the increasing global fresh water demand. This paper explores the status of the desalination industry and research work in South Korea. Desalination plant designs, statistics, and the roadmap for desalination research were analyzed. To reduce energy consumption in desalination, seawater reverse osmosis (SWRO) has been intensively investigated. Recently, alternative desalination technologies, including forward osmosis, pressure-retarded osmosis, membrane distillation, capacitive deionization, renewable-energy-powered desalination, and desalination batteries have also been actively studied. Related major consortium-based desalination research projects and their pilot plants suggest insights into lowering the energy consumption of desalination and mitigation of the environmental impact of SWRO brine as well. Finally, considerations concerning further development are suggested based on the current status of desalination technology in South Korea.

## 1. Introduction

Although there is enough fresh water to meet demands at the global level on an annual basis, spatial and temporal variations of water demand and availability lead to water shortage problems in several parts of the world [1]. Presently, almost one-half of the global population, 3.6 billion people or 47%, faces water scarcity in at least one month of the year. This number is predicted to rise to 57% of the global population by 2050 [2]. In response to steadily increasing water demand due to population growth, urbanization, and increased industrial and agricultural activities, seawater desalination has received the most attention as a viable option to address shortages and meet demand. Accordingly, there has been rapid growth in the desalination market, with an annual growth rate of almost 9% in 1990–2018 [3].

Before 2000, when thermal desalination was the main technology in the seawater desalination market, South Korean companies occupied a large portion of the seawater desalination market. However, government support for research and development on seawater reverse osmosis (SWRO) technologies was necessary to maintain the market share, as desalination was dominated by reverse osmosis (RO) technology in the 2000s. After the South Korean government selected seawater desalination technology as one of the top 10 priority research and development (R&D) projects (Value Creator 10) in 2006, desalination research has been extensively investigated in South Korea [4].

In this review article, we focused on innovations in and evolution of desalination technology in South Korea. This review aims to share the history of desalination technology in South Korea and provide insights into the development direction of the desalination industry and its research targets. Along with major global trends, the current status of the desalination industry and relevant major desalination research projects in South Korea are introduced. Reviewing the desalination technology in South Korea offers information for understanding industrial needs and a basis for further desalination research.

## 2. Fresh Water Resources and the Desalination Industry in South Korea

### 2.1. Fresh Water Resources and Desalination Facilities in South Korea

South Korea is located in a relatively wet region. The total amount of water resources in South Korea is 132.3 billion m^3^/year (2015), and the average annual precipitation in South Korea is 1277 mm (1978–2007), 1.6 times higher than the global average. However, due to the high population density, the annual precipitation per capita is 2629 m^3^ per year, only approximately 1/6 of the global average [5]. Moreover, the seasonal distribution of precipitation in South Korea is not uniform. Annual precipitation in the spring is only approximately 15%, while 40–60% of the annual total is concentrated in the summer from June to August. Most of the precipitation in winter is due to snowfall, except for the southern coastal regions, and the amount of annual precipitation in winter is 5–10% [6]. Unfortunately, these spatial and temporal deviations of available water by season are expected to become even greater due to the ever-intensifying effects of climate change and the occurrence and concentration of extreme rainfall [7].

Recently, temporal water shortage issues during the dry season have triggered the consideration of seawater desalination as a solution for a stable water supply in South Korea. For example, in 2015, Chungcheongnam-do, located in the midwestern part of South Korea, suffered severe water scarcity. To solve this problem, in 2021, the South Korean government decided to build a 100,000 m^3^/day SWRO desalination plant to supply water resources for a nearby industrial cluster [8].

In South Korea, medium-sized (5000–60,000 m^3^/day) RO desalination facilities were first built in the late 1980s to supply industrial water [8,9]. Table 1 lists representative RO desalination plants in South Korea. Most of the RO desalination facilities are brackish water desalination plants; the first seawater RO desalination plant for industrial water production was constructed in 2014. Because the water shortage problem has not been serious in most areas of South Korea, small-scale (<5000 m^3^/day) RO desalination facilities for municipal water have so far been installed only in islands. As of 2014, there are 109 seawater RO desalination facilities on islands of South Korea, with a total facility capacity of 8333 m^3^/day [10]. Ninety-seven percent of the RO desalination facilities on islands have capacities of less than 500 m^3^/day (Table 2). Because these small-scale desalination plants are located on remote small islands, proper operation and maintenance (O&M) by expert operators is difficult. As a result, some small-scale desalination plants have been closed due to O&M issues [11]. Therefore, automated O&M devices or remote-control systems should be applied for effective management.

### 2.2. Overview of Desalination Technology Trends and the Desalination Industry in South Korea

**Figure 1:**
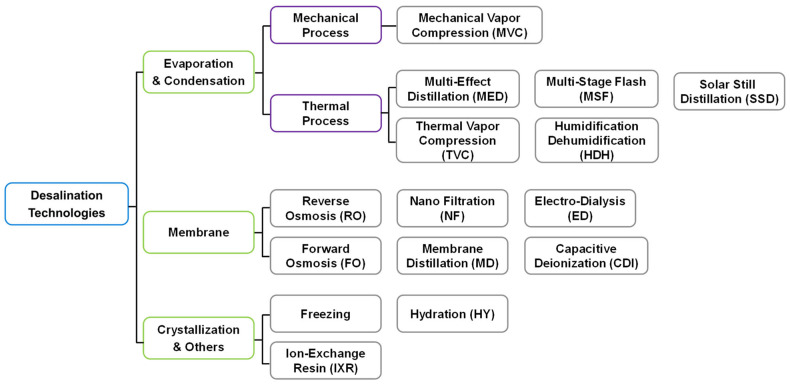
Categories of desalination technologies.

Generally, desalination technologies can be classified into three main categories: evaporation and condensation, membrane process, or crystallization. Figure 1 shows current desalination technologies, including new technologies such as forward osmosis (FO), membrane distillation (MD), and capacitive deionization (CDI) [12,13]. Evaporation technologies began operation during the 1950s and had been the primary technology until 2000 [14]. However, tremendous improvements in RO membrane technology and energy cost increases in the last years have induced a primary desalination technology switch from thermal processes to membrane-based desalination [15]. Currently, the specific energy consumption (SEC) of RO seawater desalination, 3.5–4.5 kWh/m^3^ [16], is significantly lower than that of thermal desalination technology such as multi-stage flash (MSF; 13.5–25.5 kWh/m^3^) and multi-effect distillation (MED; 6.5–11 kWh/m^3^) [13]. As a result, RO desalination (51% of new seawater desalination capacity in 2001) increased to 75% in 2003 and has continued to increase ever since [14,17].

A representative South Korean desalination plant supplier is Doosan Heavy Industries & Construction (Changwon, Korea). Doosan Heavy is a global leading seawater desalination plant supplier, occupied fifth place for desalination capacity contracted from 2010 to 2021 [18]. Doosan Heavy was mainly focused on MSF and MED desalination, but to increase its market share in the RO technology-oriented seawater desalination market, they participated in a South Korean government R&D program for RO technology from 2007 to 2012. Doosan Heavy constructed the world’s largest desalination plant, Ras Al Khair desalination plant, with a capacity of 1,036,000 m^3^/day in 2014 (MSF 727,130 m^3^/day and RO 309,360 m^3^/day) [19]. GS Engineering & Construction (GS E&C) (Seoul, Korea) is also one of the leading companies in the desalination business. In 2012, GS E&C entered the desalination business in earnest by acquiring Inima OHL, an RO desalination plant supplier in Spain [20]. The company ranked 45th among the world’s 50 largest water-related companies, and at the end of 2020, GS Inima won the desalination project with about 2.1 billion USD in Oman [21]. Engineering, procurement, and construction (EPC) companies such as Hanwha E&C (Seoul, Korea) and Daewoo E&C (Seoul, Korea) have also participated in national research projects (KORAE, 2016–2020) to develop seawater desalination technology [22].

Because the water shortage problem in South Korea has not been serious, South Korean companies are paying more attention to the global desalination market. The global water desalination equipment market size was estimated at 13.12 billion USD in 2020 and is expected to expand at a compound annual growth rate of 7.1% from 2020 to 2028 [23].

## 3. Status of Desalination Research in South Korea

### 3.1. Roadmap of Desalination Research in South Korea

**Figure 2:**
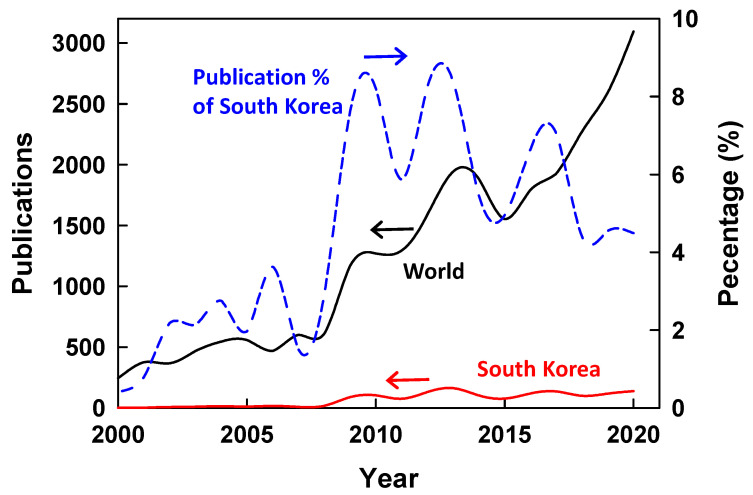
Trends of scientific publications related to desalination globally and from South Korea.

Research publication data were collected from the Scopus database using the search term desalination for the years from 2000 to 2020 [24]. By 2020, there were 26,617 global publications, of which 1447 were from South Korea, accounting for 5.4%. According to a recent bibliometric study, the number of desalination publications from South Korea ranks fourth in the world after China, the United States, and India [25]. Figure 2 shows the evolution of annual publications related to desalination worldwide and from South Korea between 2000 and 2020. The annual publications sharply increased in 2009 from 20 to more than 80 and reached its maximum of 163 in 2013. Since then, the publication numbers have fluctuated but maintained higher than 100. However, the publication percentages from South Korea have shown a decreasing trend since 2013, from 8.4% to 4.5% in 2020.

The top 20 keywords in desalination publications from South Korea are listed in Table 3. The top 10 keywords were as follows: *membrane*, *seawater*, *reverse osmosis*, *water filtration*, *water treatment*, *fouling*, *osmosis*, *wastewater treatment*, *electrode*, and *energy efficiency*. Most of the top 10 keywords are interpreted as related to RO technology, reflecting the recent RO-oriented market. And *electrode*, the ninth-ranked keyword, is considered to be related to capacitive deionization technology. The keywords ranked 11th to 20th include *capacitive deionization*, *membrane distillation*, and *forward osmosis*, which are alternative desalination technologies, showing the trend of desalination research in South Korea toward low-energy desalination.

**Figure 3:**
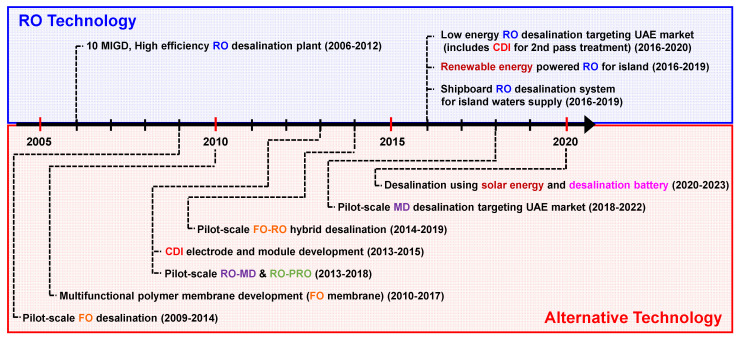
The milestones of major desalination research in South Korea.

Major desalination R&D projects in South Korea were analyzed using the National Science and Technology Information Service of South Korea (NTIS) databases [22]. The databases were searched using the keyword “desalination” and filtered for research funding of 100 million won (KRW)/year (≈85,000 USD/year) or more by 2021. According to the databases, 695 desalination research projects have been conducted since 2002. Figure 3 illustrates the major consortium-based desalination research projects in South Korea. Excellent South Korean universities, national research institutes, and major companies participated in these consortium-based research projects. As shown, the major desalination research projects can be divided into two categories, i.e., conventional RO-based desalination and alternative desalination, including FO, MD, pressure-retarded osmosis (PRO), CDI, and desalination battery. Seawater desalination research has been booming since the Center for Seawater Desalination Plant (CSDP), funded by the South Korean government, was established in 2006. After the seawater engineering and architecture of high-efficiency RO (SEAHERO) R&D project (2007–2012) was launched by the CSDP for the development of SWRO plant technologies, desalination research projects based on new alternative desalination technologies were initiated between 2009 and 2014. Although these alternative desalination research projects have shown technical feasibility using pilot-scale tests, they have not yet developed into actual large-scale (>60,000 m^3^/day) desalination plants. New research projects based on RO desalination technology with high potential for practical use have been carried out since 2016. The detailed status of each desalination technology is highlighted in what follows.

### 3.2. Reverse Osmosis

With the development of energy recovery devices (ERD) in the 1990s, the SEC of SWRO reduced from 20 kWh/m^3^ to 2–5 kWh/m^3^ [26,27], making it an energy-efficient system compared to thermal desalination technologies. In addition, the continuous innovation and improvement of SWRO membranes and plant technologies led to the steady growth in both the number and capacity of RO plants. Since 2000, almost all new desalination plants with a production capacity of over 100,000 m^3^/day worldwide were SWRO desalination plants [17]. The current production of desalinated water from SWRO is 65.5 million m^3^/day, accounting for 69% of the desalinated water production [27].

The three main components in SWRO processes are RO membranes, high-pressure pumps, and the ERD system. Membrane manufacturing companies have led the development of RO membranes in South Korea. Woongjin Chemical, a South Korean company, developed a polyamide (PA) RO membrane in the early 1990s. In 2003, Woongjin Chemical ranked third in the world for manufacturing capacity of RO membrane elements, behind Dow Chemical of the United States and Hydranautics of Japan. However, Woongjin Chemical was acquired by Toray Advanced Materials of Japan in 2014. Now, LG Chem (Seould, Korea) is the only South Korean company manufacturing RO membranes. LG Chem entered the RO membrane field in 2014 by acquiring an American startup, NanoH2O (Torrance, USA). Currently, Toray (Tokyo, Japan), DuPont (Wilington, USA) (formerly Dow), Hydranautics (Nitto Group Company) (Oceanside, USA), and LG Chem occupy more than 90% of the PAT RO membranes market [17].

**Figure 4:**
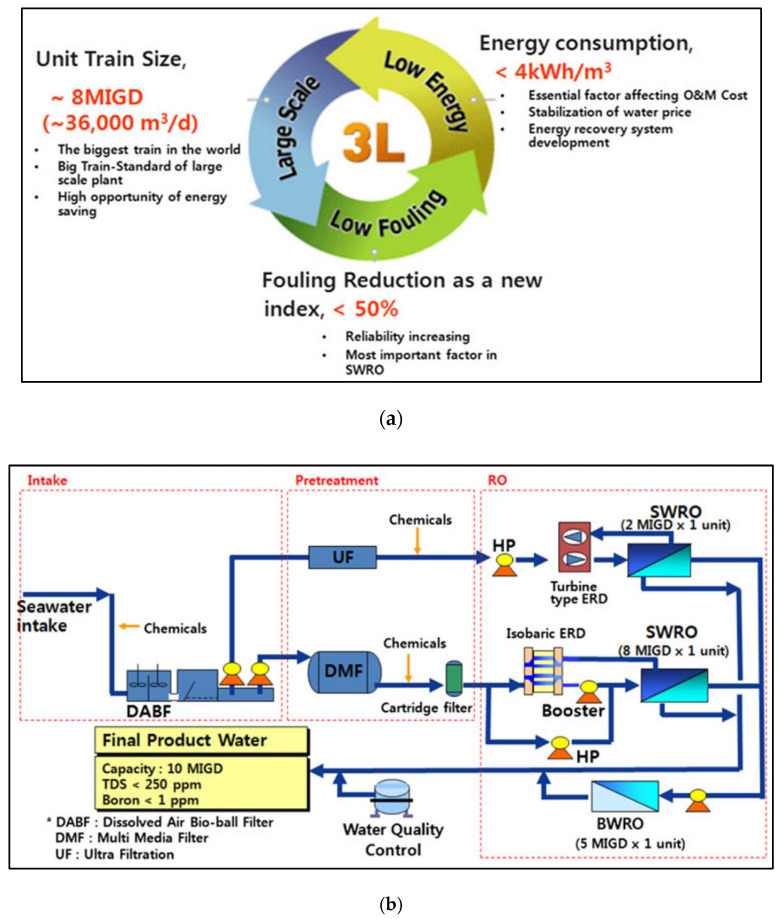
(**a**) The three main strategies in the seawater engineering and architecture of high-efficiency reverse osmosis (SEAHERO) research and development program and (**b**) the flow diagram of the SEAHERO testbed [28].

Although RO has become a major technology in the desalination market since 2000, it was only in 2007 that RO technology development actively began in South Korea with the government’s R&D support. The first consortium desalination research project, the SEAHERO R&D program, started in 2007 with 165 million USD in funding for five years to achieve world-class SWRO technology. Kim et al. introduced the technology concept for this research project in detail [28]. The research was conducted with three technical strategies and four core development technologies. The three technical strategies are low energy, low fouling, and large train size (Figure 4a). The four core development technologies consist of (I) future SWRO technology, (II) localization of core parts, (III) large-scale SWRO plant design and construction technology, and (IV) innovative O&M technology [4].

The South Korean companies Doosan Heavy, Hyosung, and Woongjin Chemical participated in the SEAHERO R&D program. They were in charge of developing design and engineering technology, a high-pressure pump, and the SWRO membrane, respectively, to achieve localization of major parts of the SWRO process. The most notable achievement of the task was an 8 million imperial gallons per day (MIGD) (≈36,000 m^3^/day) single SWRO train with dual-media filtration pretreatment. With another 2 MIGD SWRO trains using membrane pretreatment, the total water production capacity was 10 MIGD (≈45,000 m^3^/day) (Figure 4b). In addition, a 16-inch SWRO spiral-wound element was developed to reduce the initial investment cost and the operating costs of the SWRO plant. The SEC of the SWRO testbed was estimated to be 3.7 kWh/m^3^ at the feedwater temperature of 10–15 °C. Later, the high-pressure pump developed in this project led to export-worthy performance [29].

### 3.3. Forward Osmosis

Although RO technology has dramatically reduced the SEC of desalination, seawater desalination is still an energy-intensive process. Therefore, advanced desalination technologies have been investigated to reduce energy use further. One of the alternative technologies is FO membrane technology. The RO membrane process requires hydraulic pressure higher than the osmotic pressure of seawater for water transport, typically 60–70 bar. However, FO technology uses the osmotic pressure as the driving force for water transport; thus, the FO process needs much lower energy for water flux. After introducing the FO process using ammonia–carbon dioxide as the draw solute in 2005, FO has received tremendous attention in academic research and industrial development [30,31,32]. In the early 2000s, most research has focused on cellulose triacetate (CTA) membranes from Hydration Technologies Inc. However, the commercial CTA membrane showed insufficient performance, such as degradation when exposed to an ammonium bicarbonate draw solution, relatively low water permeability, and salt rejection [33]. Then, research on a specifically tailored PA thin-film composite (TFC) membrane with a porous support layer to minimize internal concentration polarization led to the introduction of a commercial TFC FO membrane, i.e., from Oasys Water in the USA in 2008 [32,34].

Up to now, three consortium-based FO research projects have been conducted in South Korea, as shown in Figure 3. The first and second projects, started in 2009 and 2010, focused on developing FO membranes. As a result, Woongjin Chemical, which participated in the first research project, succeeded in developing a commercial-level spiral-wound element by using its PA TFC RO membrane technology for mass production. The developed FO membrane was applied in FO desalination pilot tests [35,36]. Although the FO pilot testes showed better water flux performance for the developed FO membrane, the research projects faced challenges with applications of the FO process, such as the FO–RO hybrid desalination process using a divalent draw solute.

Water permeating through the FO membranes dilutes the draw solution. Thus, to obtain fresh water as a final product, an additional process for draw solute separation is required. Because of the high energy consumption of the separation process, the theoretical thermodynamic energy required for desalination with FO is always higher than that without FO [32]. Alternatively, an FO hybrid desalination process using the osmotic dilution concept can be a low-energy process [37,38,39]. Osmotic dilution means using the osmotic difference between two solutions for water permeation in FO without a draw solution separation process, resulting in a diluted draw solution as a product of the FO process.

**Figure 5:**
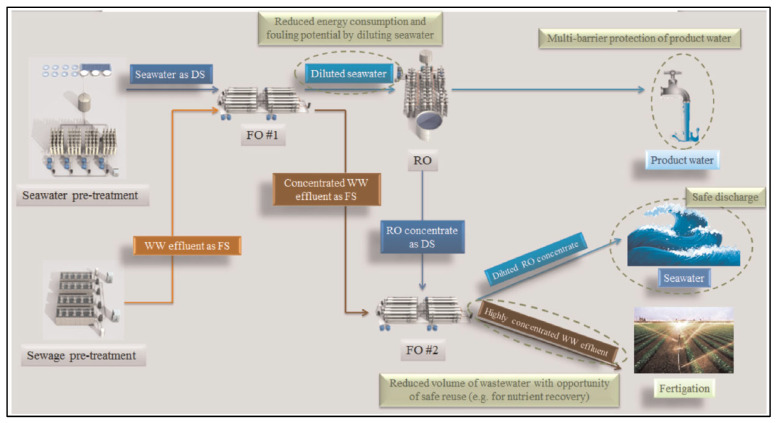
Schematic diagram of the osmotic dilution forward osmosis–reverse osmosis (FO–RO) desalination hybrid pilot plant [37].

The third FO research project that began in 2014 investigated an osmotic dilution FO–RO hybrid process using the world’s first pilot-scale plant. Figure 5 shows a schematic diagram of the FO–RO hybrid pilot plant. As shown, the effluent of wastewater treatment is used as the feed solution, and seawater is used as the draw solution. Permeated wastewater dilutes the seawater, decreasing the osmotic pressure of the seawater. Because the applied hydraulic pressure of RO depends on the osmotic pressure of the seawater, the osmotic dilution of the FO process can reduce energy consumption in the subsequent SWRO process [14,38]. The goal of the research was a reduction of the SEC of desalination to 2.5 kWh/m^3^, and the feasibility was tested in a FO–RO hybrid pilot plant with a capacity of 1000 m^3^/day [40]. The pilot plant adapted the plate-and-frame type FO membrane module from Porifera. According to the modeling estimation, the SEC of the FO–RO hybrid desalination plant was 2.16 kWh/m^3^, which is 24.7% lower than the SEC of the SWRO process, which is 2.87 kWh/m^3^ [41].

### 3.4. Pressure-Retarded Osmosis

Unlike RO or FO processes, which are primarily intended for water treatment by membrane separation, the purpose of the PRO process is to obtain energy from a salinity gradient. PRO uses osmotic water transport, similar to FO, but a hydraulic pressure less than the osmotic pressure difference is applied on the higher concentration solution. The water flux is decreased due to the hydraulic pressure, but energy generation occurs when releasing the hydraulic pressure through a turbine [42]. The theoretical feasibility of PRO was investigated in the 1950s and 1960s, though the development of PRO has been limited due to the complexity of the system and the lack of a suitable membrane [42,43]. The world’s first prototype PRO power plant was built by a Norwegian company, Statkraft, in 2009 [42]. Practical electricity generation is still economically challenging because of the insufficient PRO membrane performance. Based on the Statkraft analyses, the minimum power density performance of the membrane should be at least 5 W/m^2^ for a commercially viable PRO process [44,45]. Instead of electricity generation, the Mega-ton Water System project in Japan investigated the PRO process to produce hydraulic pressure using a hydraulic Pelton turbine in 2010 [46]. In this project, the PRO pilot plant used 460 m^3^/day of SWRO brine as the draw solution and 420 m^3^/day of treated wastewater as the feed solution [47]. A hollow-fiber PRO membrane, developed by the Toyobo company, was applied, and the self-reported minimum power density was more than 12 W/m^2^, at approximately 30 bar applied hydraulic pressure [46].

**Figure 6:**
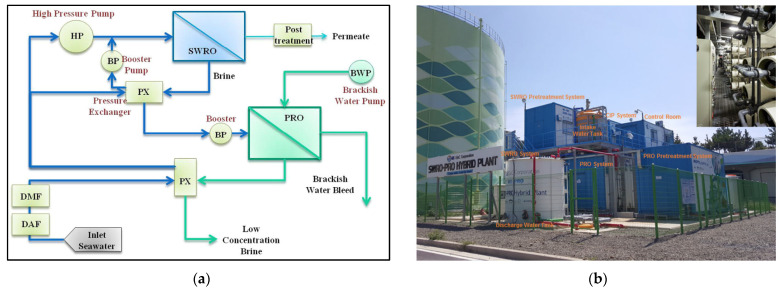
(**a**) Schematic diagram of osmotic dilution seawater reverse osmosis–pressure-retarded osmosis (SWRO–PRO) desalination hybrid pilot plant, (**b**) reverse osmosis (RO)–PRO hybrid desalination pilot with SWRO water production capacity of 240 m^3^/day [46,48].

The technical challenges of desalination are the relatively high economic costs and environmental concerns caused by brine [14]. To solve these issues, the Global Membrane distillation, Valuable source recovery, PRO (MVP) program, a South Korean government-funded project, evaluated the feasibility of RO–PRO and RO–MD hybrid processes for energy recovery and SWRO brine treatment between 2013 and 2018. An RO–PRO hybrid desalination pilot plant with a water production capacity of 240 m^3^/day was constructed and operated for more than two years to verify the long-term operation capability [47]. The developed pilot plant used SWRO brine and reclaimed wastewater for the PRO process, and two isobaric pressure exchangers were adopted to use hydraulic pressure without converting it to electricity (Figure 6) [46]. A spiral-wound PRO membrane was developed by Toray Advanced Materials, and GS E&C was responsible for developing the PRO engineering technology. The PRO process dilutes the SWRO brine to mitigate its adverse impact on the marine environment, and the hydraulic pressure from the salinity gradient reduces the SEC of the SWRO process simultaneously. According to the pilot plant test, the SEC was reduced by 19%, and the SWRO brine was diluted to 63% of its original concentration by applying the PRO process [47]. To improve the SWRO–PRO technology, the development of PRO elements with higher recovery and the optimization of cleaning techniques for PRO membranes are required [45,47,48].

### 3.5. Membrane Distillation

MD is a thermal, membrane-based desalination process driven by the vapor pressure difference across a hydrophobic, microporous membrane. MD is more energy-intensive than RO. The minimum theoretical energy required for seawater desalination by single-pass direct contact MD and RO is 27.6 MJ/m^3^ and 3.8 MJ/m^3^, respectively [49]. However, MD has drawn attention as an emerging technology because the MD process can be operated with low-grade heat sources, including solar energy, geothermal energy, and waste heat energy from industrial production [50]. Moreover, MD can treat high-salinity water that cannot be desalinated by RO.

**Figure 7:**
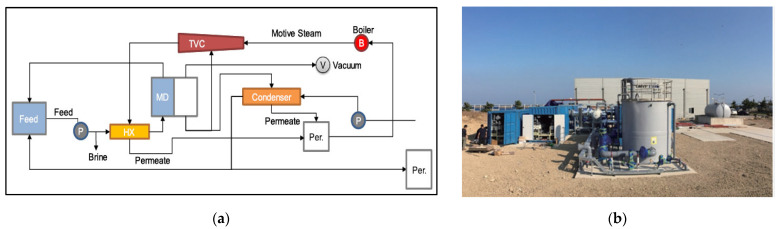
(**a**) Schematic diagram of membrane distillation (MD) desalination system, (**b**) MD desalination pilot plant with a water production capacity of 400 m^3^/day [52,54].

MD technology for SWRO bine treatment was one of the research themes in the Global MVP project. SWRO brine has total dissolved solids (TDS) concentrations of approximately 70,000 mg/L. Consequently, the hydraulic pressure required to overcome the osmotic pressure of the brine can be greater than the maximum allowable pressure of the SWRO membrane modules and other process equipment [14,51]. The goal of the SWRO–MD research theme was to reduce the volume of brine by 30% [52]. Previous pilot-scale MD plants used flat or spiral-wound type modules, but the Global MVP project MD pilot plant used hollow-fiber MD modules developed within the research project by Econity Inc. (Yongin, Korea) [52]. Figure 7 shows the process flow diagram of the vacuum MD pilot plant with thermal vapor compression. In the initial stage, an MD pilot plant with a scale of 10 m^3^/day was built, and the performance of the MD membrane module and the influence of operating conditions were evaluated [53]. In addition, energy optimization of the MD process was conducted by combining it with renewable solar energy [52]. Later, a larger MD pilot plant with a capacity of 400 m^3^/day was designed for SWRO brine treatment [54]. With these development achievements, the next MD research project targeting the desalination market in Middle Eastern countries was launched in 2019. The MD pilot plant will be constructed in a Middle Eastern country and will focus on developing O&M technologies and the use of renewable energy [22].

### 3.6. Capacitive Deionization

Recently, capacitive deionization (CDI) technology has been extensively studied as an emerging desalination technology. CDI is an electrosorption technology to deionize water by applying an electrical potential difference (usually <1.2 V) over two electrodes, which are often made of porous carbon [55]. The ions are temporarily adsorbed on the surface of the charged electrodes with an external DC energy source, and the adsorbed ions are released back into the solution by short-circuiting or reducing the voltage across the electrodes. In the same manner as a capacitor, energy can be partially recovered during the electrode regeneration step [56,57,58]. The energy consumption in the CDI process strongly depends on the salt concentration of the feed water because CDI desalinates ions by electrosorption [59]. Thus, it has been suggested that CDI is more energy-efficient than RO for brackish water desalination when the feed salinity is lower than 3.5 g/L, and the product water salinity is 1 g/L [60].

**Figure 8:**
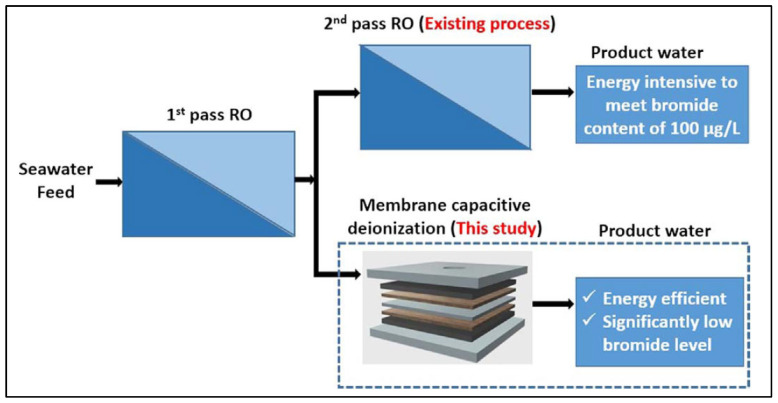
Conceptual diagram of reverse osmosis–capacitive deionization (RO–CDI) process replacing second-pass RO with CDI [55].

The pilot-scale CDI process has been investigated under themes of the South Korean Optimized RO desalination integrated with the Advanced Energy Saving (KORAE) research project from 2016. The most critical goal of the KORAE project is to develop SWRO technology to reduce energy consumption. SWRO technology is a mature technology, leaving little room for improvement in terms of energy consumption. One of the technical approaches for reducing energy consumption in the project is modifying a second-pass RO process. A second-pass RO is used either when the source seawater salinity is higher than 35,000 mg/L or when stringent final water quality standards must be met for items such as TDS or boron [61]. Boron is one of the most difficult seawater components to remove because boron naturally exists as the undissociated neutral form of boric acid (pK_a_ 8.9) at the typical pH of seawater (8.1–8.2) [62]. As a result, many SWRO plants have been designed with a two-pass array for boron removal, and the energy consumed in a second-pass RO is typically 0.5 kWh/m^3^ [63,64,65]. Therefore, if the CDI process can remove boron or TDS using less than 0.5 kWh/m^3^, some energy savings can be achieved. Figure 8 shows the conceptual process flow of an SWRO–CDI process replacing the second-pass RO with CDI [55]. A pilot-scale Membrane CDI (MCDI) process with a capacity of 50 m^3^/day, developed by Siontech, was examined as an alternative process to the second-pass RO. The CDI process treated the permeate from a single-pass SWRO pilot system (120 m^3^/day). The energy consumption of the MCDI process was estimated as less than 0.4 kWh/m^3^ with an SWRO permeate concentration of less than 500 mg/L [22]. Moreover, the developed MCDI process can be used to remove not only boron and TDS but also bromide, a precursor of bromated disinfection byproducts [55]. Based on lab-scale tests regarding bromide removal, the energy consumption was estimated to be between 0.05 kWh/m^3^ and 0.3 kWh/m^3^, depending on the TDS of the feed water [55]. The energy consumption of CDI can be further optimized by achieving energy recovery from the CDI and using a larger, full-scale CDI process. The KORAE project plans to build an SWRO pilot plant with a capacity of 1000 m^3^/day in the United Arab Emirates (UAE). Accordingly, the CDI process will also be further examined in terms of energy consumption, removal efficiencies, and O&M [66].

### 3.7. Renewable-Energy-Powered Desalination

Energy consumption is a major factor affecting water production costs. In the case of seawater desalination, energy consumption contributes 20–35% of the total costs of water production, and it can exceed 50% in extreme conditions for remote plant locations with high unit energy costs [67]. To mitigate this issue, renewable energy use has been suggested as an option to reduce desalination energy requirements. In addition, energy consumption savings can reduce greenhouse gas emissions. The other motivation for developing renewable-energy-powered desalination is recent policies in the Middle East countries regarding the post-oil era [68]. The post-oil projects include renewable-energy-powered desalination. For example, Masdar, the renewable energy company in the UAE, launched a renewable energy desalination pilot program in 2013 to develop advanced technology for sustainable water production [69]. Moreover, the world’s largest solar desalination plant recently built in Al Khafji City, Saudi Arabia, with a capacity of 60,000 m^3^/day, demonstrates the commitment of the Middle East to the post-oil era.

Renewable-energy-powered desalination has high growth potential, but it is still in the early stages of its application. Currently, installed renewable-energy-powered desalination plants account for only 1% of global desalination capacity [70,71]. The technically and economically available renewable energy significantly varies by country. The majority of renewable-energy-powered desalination uses wind, solar, or geothermal energy, and the most dominant combination is photovoltaic (PV)-powered RO systems [72]. For small-scale desalination in remote areas, a standalone renewable-energy-powered desalination facility is beneficial considering the high cost of grid connectivity [73].

In South Korea, several renewable-energy-powered desalination facilities have been installed in connection with energy self-sufficient island projects. For instance, a small RO desalination plant with a solar PV energy and energy storage system (ESS) was built on Juk-do island for 70 residents [74]. As a similar energy self-sufficient island project, a PV–RO plant was built on Soijak-do island to supply energy and fresh water for 114 residents with a capacity of 200 m^3^/day. The plant is equipped with a 100 kW solar power system, 300 kWh ESS, water quality monitoring and remote-control facilities. Although renewable-energy-powered seawater desalination technology has not been widely applied yet, it will be continuously researched to meet the demand in major desalination markets like the Middle East countries and address water shortages on small islands like those in South Korea.

### 3.8. Desalination Battery

Desalination batteries (or seawater batteries) are categorized as an electrochemical desalination process. Desalination battery removes salts by adsorption of dissolved ions, such as electro-deionization and capacitive deionization. A desalination battery has been considered a more appropriate electrochemical desalination technology because of the high salt adsorption capacity (SAC) with high-capacity battery electrode materials [75]. Due to its limited SAC, CDI has been used only for brackish water desalination.

The desalination battery operating process is based on the electrochemical redox reaction. The system consists of the feed or seawater as the electrolyte with Na- and Cl- storage electrodes, which remove salt during the charging process. When charging the system, sodium and chloride ions in the seawater are attracted into the Na-storage electrode and Cl-storage electrode, respectively, resulting in desalination. However, during discharge, ions are released, and the electrodes are regenerated. After the desalination battery was introduced in 2012, it emerged as a next-generation desalination technology [76]. In the past decade, various types of desalination batteries have been developed to enhance desalination capacity, including rocking chair, redox flow, and metal-air desalination batteries [77,78].

Ulsan National Institute of Science and Technology (UNIST) in South Korea is a global leader in desalination battery technology. UNIST developed a battery based on a solid electrolyte. The Korea East–West Power company (KEWP) (Ulsan, Korea) and Korean Electric Power Corporation (KEPCO) (Naju, Korea) are doing research together with UNIST to develop 10 kWh electrical energy storage with desalination batteries. KEWP and KEPCO invested approximately 4,200,000 USD in UNIST during 2016–2019 [79]. Recently they introduced the concept of seawater desalination by rechargeable seawater batteries [80,81]. This type of desalination battery is composed of an open cathode, a sodium super-conducting separator which is the solid electrolyte, and a closed metal-organic anode. In addition, they compared the rechargeable seawater battery desalination system with the RO desalination system.

## 4. Conclusions and Prospects

This review summarizes the current status and trends of desalination technology in South Korea. Similar to global desalination technology trends, the key direction in developing desalination technology in South Korea is reduced energy consumption. In South Korea, significant research on desalination technology has been actively carried out since 2006. As a result, the number of scientific publications on desalination from South Korea ranks fourth in the world after China, the United States, and India. The most studied research topic is RO membrane desalination, and emerging technologies including FO, PRO, MD, CDI, renewable-energy-powered desalination, and desalination batteries have also been actively studied. It is noteworthy that feasibility studies for the emerging technologies have been carried out on a pilot scale. Table 4 summarizes the major consortium-based desalination research projects and their pilot plants. These studies focused on lowering the energy consumption of desalination and mitigating the environmental impacts of SWRO brine as well.

Research into SWRO desalination technologies has led to the localization of important components of the SWRO process, such as RO membranes and high-pressure pumps. However, there are still ample research needs for the localization of technologies, including high-efficiency ERD, pretreatment, and posttreatment in the SWRO plants.

In South Korea, most SWRO facilities for municipal water are installed on islands with small capacities of less than 500 m^3^/day. The need for small desalination facilities on remote small islands will continue to increase in the future, considering the effects of climate change. The primary limitations to further applications of small SWRO facilities are the O&M of the system and economic feasibility. Automated O&M units or remote-control systems will be necessary to allow effective management of small SWRO facilities on islands. SWRO plants with renewable energy sources can reduce the economic costs of desalination.

Due to the abundance of fresh water and the low cost of tap water, medium and large SWRO plants have been considered only for industrial water production in the midwestern part of South Korea, which experiences seasonal water scarcity. Desalination can be the best approach to provide stable fresh water for industry and agriculture in these water-scarce areas. The development of SWRO membranes that can meet water quality requirements using single-pass SWRO can also reduce the total cost of SWRO desalination. As the number of reference plants demonstrating performance in terms of water quality and process stability increases, opportunities for other applications such as power and municipal sectors will develop.

Globally, almost half of the desalination capacity is located in the Middle East and North Africa (MENA) region [82]. Accordingly, South Korean companies promote the construction of large-scale desalination plants in overseas markets rather than domestic markets. To increase orders for large-scale desalination plants, it is necessary to develop SWRO engineering technologies and O&M technologies optimized for the MENA region, i.e., pretreatment technologies for severe algal blooms or accidental oil spills. Pilot-scale studies conducted in the MENA region can provide practical information for developing optimal O&M technologies to meet local requirements.

A major environmental concern of desalination is the disposal of the brine produced in the desalination process. Hybrid RO technology processes with emerging technologies such as dilution FO–RO, RO–PRO, and RO–MD not only can resolve the brine issues but also can create new desalination markets. Through pilot-scale research on these technologies, major progress has been made in developing membranes and engineering technologies. However, considering the current technical status and the small market for emerging technologies, continuous support by government policy and additional research and demonstrations are required to improve the performance for commercialization.

## Figures and Tables

**Table 1:** Representative RO desalination facilities in South Korea.

Plant Location	Capacity (m^3^/day)	Feed Water Type	1st Year of Production	Purpose
Seosan	16,000	brackish water	1988	industrial water
Seosan	25,000	brackish water	1990	industrial water
Seosan	84,000	brackish water	1991	industrial water
Dangjin	4500	brackish water	1997	industrial water
Dangjin	182,000	brackish water	2009	industrial water
Daesan	119,000	brackish water	2012	industrial water
Gwangyang	30,000	seawater	2014	industrial water
Samcheok	2400	seawater	2017	power plant
Uljin	10,000	seawater	2020	power plant
Daesan	100,000	seawater	2024 (plan)	industrial water

**Table 2:** Capacity of seawater RO desalination facilities for municipal water on islands of South Korea.

Capacity (m^3^/day)	Number of Facilities	Percentage (%)	Purpose
10–49	60	55.0	municipal water
50–99	29	26.6	municipal water
100–499	17	15.6	municipal water
500–1000	3	2.8	municipal water

**Table 3:** Top 20 keywords for desalination research in South Korea.

Rank	Keywords	Number
1	membrane, membranes, membrane technology	569
2	seawater, seawater desalination, sea water	522
3	reverse osmosis, seawater reverse osmosis, RO membrane, reverse osmosis desalination	377
4	water filtration, filtration	377
5	water treatment, water purification, purification	275
6	fouling, membrane fouling, fouling control	226
7	osmosis	182
8	wastewater treatment, wastewater, wastewater reclamation, waste water management	162
9	electrode, electrochemical electrode	159
10	energy efficiency, energy consumption, specific energy consumption	152
11	capacitive deionization, membrane capacitive deionization	150
12	distillation	148
13	water, water supply	143
14	sodium chloride	119
15	forward osmosis	115
16	membrane distillation, direct contact membrane distillation	115
17	energy utilization	105
18	concentration	94
19	polarization, concentration polarization	93
20	biofouling, biofilm	89

**Table 4:** Summary of representative desalination research programs and their pilot plants in South Korea.

Research Program	Period	Technology	Pilot Scale (m^3^/day)	Major Achievements and Features
SEAHERO program	2007–2012	SWRO	45,000	SWRO testbed 16-inch SWRO element high-pressure RO pump
Development of multi-purpose FO desalination plant	2009–2014	FO	20	spiral-wound FO element FO–RO hybrid pilot plant
Global MVP program	2013–2018	PRO MD	240 (PRO) 400 (MD)	PRO pilot plant with pressure exchanger spiral-wound PRO element MD pilot plant MD membrane
FOHC program	2014–2019	FO	1000	osmotic dilution FO–RO hybrid pilot plant
KORAE	2016–2020	RO CDI	120 50	high flux and high-efficiency RO membrane for single-pass SWRO system MCDI module SWRO–CDI pilot plant SWRO pilot plant in UAE (1000 m^3^/day) (plan)
